# Development of Cortisol Sensors with Interdigitated Electrode Platforms Based on Barium Titanate Nanoparticles

**DOI:** 10.3390/s25113346

**Published:** 2025-05-26

**Authors:** Marylene S. G. Roma, Juliano A. Chaker

**Affiliations:** 1 Federal Institute of Brasilia, Ceilândia Campus, Brasília 72220-900, DF, Brazil; 2 Department of Biomedical Equipment Technician, University of Brasília, Ceilândia Campus, Brasília 72220-900, DF, Brazil; 3 Laboratory of Nanoscience and Nanotechnology, University of Brasília, Ceilândia Campus, Brasília 72220-900, DF, Brazil

**Keywords:** biosensor, interdigitated electrode, barium titanate nanoparticles, electrochemical impedance spectroscopy, BET

## Abstract

Cortisol is a key biomarker for stress detection, and its levels can be monitored using point-of-care devices with sensors such as nanoparticles and interdigitated array electrodes (IDEs). This study developed an IDE platform using barium titanate (BaTiO_3_) particles synthesized via colloidal precipitation with titanium tetraisopropoxide, barium chloride, and Pluronic^®^ P123. The calcination temperatures varied between 160 °C and 340 °C, with optimal results observed at 160 °C. Scanning electron microscopy revealed particles with an average size of 26 nm, and Fourier transform infrared spectroscopy confirmed the molecular composition after the removal of P123. X-ray diffraction analysis revealed anatase and brookite phases. Brunauer-Emmett-Teller analysis indicated changes in pore morphology, with samples treated at 160 °C exhibiting a type IV(a) mesoporous structure, a surface area of 163 m^2^/g, and an average pore diameter of 5.24 nm. Higher temperatures led to transitions to type IV(b) at 260 °C and type V at 340 °C, with reduced pore size. Electrochemical impedance spectroscopy was employed to evaluate the performance of the IDE sensor integrated with BaTiO_3_ nanoparticles and albumin across cortisol concentrations ranging from 5.0 to 20 ng/mL. Impedance measurements revealed a significant decrease in impedance (Z′) with increasing cortisol concentrations, indicating increased conductivity. Specifically, Nyquist plots for a saliva sample containing 5 ng/mL cortisol—within the typical physiological range—exhibited a marked increase in charge-transfer resistance (Rct), confirming the sensor’s ability to detect low hormone levels in biological fluids. These findings underscore the potential of BaTiO_3_-based IDE platforms at 160 °C for stress biomarker monitoring.

## 1. Introduction

### 1.1. Cortisol Monitoring

Cortisol is a critical hormone for maintaining physiological homeostasis, released in response to stressful stimuli through the activation of the hypothalamic-pituitary-adrenal (HPA) axis. It plays a central role in orchestrating the “fight-or-flight” response, modulating metabolic pathways, immune function, and emotional regulation. However, prolonged exposure to elevated cortisol levels has been associated with deleterious effects, including immunosuppression, increased susceptibility to cardiovascular diseases, psychiatric disorders, and chronic fatigue. In this context, the accurate quantification of cortisol serves as a valuable tool for monitoring physiological balance, given its regulatory role across multiple metabolic, immunological, and neuroendocrine pathways [1].

Conventional cortisol sampling methods vary in terms of accuracy, feasibility, and suitability for real-time monitoring. Cortisol is present in all major biofluids, including sweat, saliva, interstitial fluid, and blood (serum), offering a broad spectrum of biological matrices for sensor development. Each matrix presents distinct advantages and limitations that directly impact its applicability. Blood (serum or plasma) is considered the gold standard for clinical diagnostics, as it allows the quantification of both free and protein-bound cortisol, ensuring the high analytical precision and rapid detection of hormonal fluctuations. However, its invasive collection can induce stress in patients, skewing cortisol levels [2].

Among alternative matrices, sweat has emerged as a promising candidate for wearable sensing technologies because of its noninvasive nature and strong correlation with circulating cortisol levels. Nonetheless, its erratic secretion pattern presents practical limitations for continuous sampling, often requiring stimulation through physical activity or iontophoresis. Interstitial fluid, which can be accessed via techniques such as microdialysis or microneedles, represents another viable medium for continuous monitoring, as it reflects the concentration of free cortisol and can be sampled even during sleep. Additionally, saliva stands out as a cost-effective, noninvasive, and physiologically relevant matrix that accurately mirrors the biologically active fraction of cortisol. Its ease of collection and suitability for repeated sampling throughout the day make it particularly attractive for rapid tests and the dynamic monitoring of circadian hormonal variations [2].

### 1.2. Biosensors

To address the growing demand for precise diagnostics and personalized therapies, technological advancements have increasingly shifted from conventional laboratory tests toward digital systems based on noninvasive biological samples. These innovations enable more accurate diagnostics and real-time therapeutic adjustments. The development of biosensors aims to enhance the sensitivity, accessibility, and personalization of physiological monitoring, with considerable potential for application across diverse clinical and environmental contexts [1].

Although promising, current electrochemical biosensors for stress biomarkers have notable limitations. Systems, for example, based on antibodies or aptamers, such as those reviewed by [3], are costly, become less stable over time, and require invasive sampling. The multiplexed device reviewed by [4], despite enabling simultaneous cytokine detection, relies on complex electronics and calibration, limiting its practicality for portable point-of-care applications.

In contrast, the sensor proposed in this work demonstrates distinct advantages. First, it provides the reliable electrochemical detection of cortisol at 5 ng/mL in real human saliva, which coincides with the average physiological level, validating its applicability in clinical conditions. Second, the interdigitated electrode (IDE) platform used is planar, scalable, and compatible with POC miniaturization. Third, the hybrid functional layer—comprising mesoporous BaTiO_3_ nanoparticles, bovine serum albumin (BSA), and a urethane-siloxane polymer—offers a high surface area, dielectric performance, and selective biomolecular adsorption. Fourth, the BaTiO_3_ calcination-tunable mesostructure enhances interface conductivity and sensitivity, and this sensing architecture is modular and chemically adaptable, making it suitable for the future detection of non-steroidal stress-related biomarkers such as α-amylase, DHEA, and inflammatory cytokines, supporting the development of multiplexed stress monitoring systems in next-generation portable diagnostics.

### 1.3. BaTiO_3_

Morphological control at the mesoscale has enabled the development of materials with enhanced properties, including tunable size, shape, surface area, interface characteristics, porosity, and the structural uniformity of mesoscopic components. Since the advent of mesoporous silica, the synthesis of novel materials using surfactants and copolymers as structure-directing agents has expanded considerably, opening new avenues in catalysis, adsorption, sensing, and electronic devices [5]. Current trends highlight two major directions: the extension of synthesis strategies to multicomponent metal oxides and the crystallization of pore walls to improve catalytic, electrical, and mechanical properties. Among these, the mesoporous metal oxide synthesis of BaTiO_3_ stands out owing to its perovskite structure and multifunctional properties—ferroelectric, piezoelectric, dielectric, and magnetoresistive—which are essential for advanced technological applications. BaTiO_3_-based materials have demonstrated continuous miniaturization and enhanced performance in devices such as multilayer ceramic capacitors (MLCCs), dynamic random-access memory (DRAM), optical sensors, and transducers. A particularly important attribute is the high dielectric constant of BaTiO_3_ ceramics synthesized from fine powders, which can range from 1500 to 6000 at room temperature. In our study, nanostructured particles exhibited a large surface area and nanoscale dimensions, significantly enhancing their functional capabilities. Although their synthesis is inherently complex, BaTiO_3_ nanoparticles possess favorable characteristics, including biocompatibility, chemical stability, and suitability for aqueous sensing platforms, particularly for salivary cortisol detection, where they offer high sensitivity and potential for integration into miniaturized and wearable diagnostic systems [5].

The present study has significant technological relevance because it advances the detection of biomarkers—specifically cortisol—through the development of an innovative electrochemical sensor based on the integration of mesoporous barium titanate (BaTiO_3_) nanoparticles onto interdigitated microelectrodes (IDEs). The mesoporous structure of BaTiO_3_, coupled with its high dielectric constant, facilitates biomolecular interactions and amplifies the electrochemical response. The sensor was functionalized with an active hybrid layer composed of BaTiO_3_ nanoparticles, bovine serum albumin (BSA), and an organic-inorganic urethane-siloxane copolymer, forming a highly sensitive, selective, and stable electrode-sample interface [6].

The sensor’s performance was evaluated through electrochemical impedance spectroscopy (EIS), which revealed a precise and linear response to varying cortisol concentrations. The BaTiO_3_ powders employed in this study exhibited an average particle size of 26 nm and a specific surface area of 163 m^2^/g, significantly surpassing traditionally reported values [6], and these contributed to the high sensitivity of the sensing platform. The proposed architecture supports the miniaturization of the device, enabling its integration into noninvasive wearable systems with strong potential for continuous, real-time stress monitoring.

## 2. Materials and Methods

### 2.1. Materials

Titanium(IV) isopropoxide (C_12_H_28_O_4_Ti, 97%, Aldrich, St. Louis, MI, USA), ethanol (CH_3_CH_2_OH, 99%, Sigma-Aldrich, St. Louis, MI, USA), barium chloride (BaCl_2_, 99.9%, Aldrich, USA), Pluronic^®^ P123 [poly(ethylene glycol)-block-poly(propylene glycol)-block-poly(ethylene glycol)] (Sigma-Aldrich, St. Louis, MI, USA), acetic acid, and sodium acetate (NaAc) were used. The hybrid polymers 3-(triethoxysilyl)propyl isocyanate (IsoTrEOS, 95%, Sigma-Aldrich, USA), poly(propylene glycol) bis(2-aminopropyl ether) (PPO 2000, Mn ~2000, Sigma-Aldrich, USA), tetrahydrofuran (THF), and absolute ethanol were also employed. Additional reagents included BSA (pH 7, 98%, Sigma-Aldrich, SP, USA), cortisol standard (100 ng/mL, Sigma-Aldrich, São Paulo, Brazil), and ammonium hydroxide (NH_4_OH, LR, ~30% NH_3_, Vetec, Vila Galvão, SP, Brazil).

### 2.2. Active Deposit Layer

#### Synthesis of BaTiO_3_ Mesoporous Nanoparticles

To prepare the mesoporous material, 2 g of the surfactant P123 was dissolved in 56 mL of a buffer solution (pH 5.9) using a hot plate (IKA KCT Basic) set at 830 rpm and 40 °C for 45 min. A solution of the Ti(IV) precursor (3.49 mL) in absolute ethanol (4.17 mL) was prepared in a beaker and then added dropwise to the P123 solution under continuous stirring for 15 min. A separate solution was prepared by dissolving 1.25 g of BaCl_2_ in 4 mL of deionized water. The mixture was prepared and added dropwise to a glass with a lid under magnetic stirring at 830 rpm and 40 °C for 45 min.

In sol-gel synthesis, hydrolysis, and condensation reactions occur when the Ti(IV) precursor reacts with water, forming a homogeneous product. The hydrolysis rate is regulated by adjusting synthesis conditions, such as the pH and temperature. The Ti(IV) precursor underwent hydrothermal hydrolysis, condensation, and chemical reflux.

The solution was aged at 90 °C without stirring for 24 h on a hot plate, forming a white precipitate. The precipitate was isolated via centrifugation (Hitachi CR22 GIII) at 12,000 rpm and 20 °C for 7 min and washed 3 times with deionized water. The precipitate was suspended in 200 mL of 95% ethanol containing 1.27 mL of HCl in a 500 mL round-bottom flask for surfactant removal. The suspension was subjected to chemical reflux in a volumetric flask containing 200 mL of ethanol (95%) and 1.27 mL of HCl at 90 °C with magnetic stirring for 24 h.

After reflux, the precipitate was washed thrice via centrifugation using a 70% ethanol-water solution. The material was then dried under a vacuum at 40 °C for 12 h. Finally, the mesoporous powder was calcined at various temperatures, ranging between the following temperatures: 160 °C, 180 °C, 200 °C, 220 °C, 260 °C, 270 °C, 280 °C, 300 °C, 320 °C, and 340 °C for 10 h with a heating rate of 40 °C/min for the complete removal of the P123 surfactant, following a modified process [7].

### 2.3. Urethane-Siloxane Organic-Inorganic Binder

An organic-inorganic urethane-siloxane precursor polymer was synthesized as a binder to enhance the adhesion of mesoporous nanoparticles to the IDE surface. For the synthesis of the organic-inorganic polymer with a high dielectric constant, 1023.0 g of poly(ethylene glycol)-block-poly(propylene glycol)-block-poly(ethylene glycol) was dissolved in 740 mL of absolute ethanol, along with 2583.27 mL of IsoTrEOS. The reagents were combined with THF in a round-bottom flask and subjected to chemical reflux at 500 rpm for 24 h at 80 °C. After the reaction, the solvent was removed using a rotary evaporator under reduced pressure at 60 °C and 120 rpm, following a modified method adapted from [8].

### 2.4. IDE Sensor Assembly

**Figure 1:**
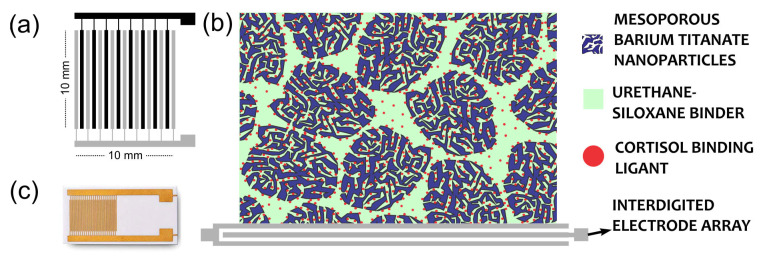
(**a**) A schematic showing the interspersed comb structure of the IDE arrangement; (**b**) the hypothetical model of nanoparticle deposition on the IDE surface; (**c**) an image of a typical IDE with Au thin film working electrodes.

The proposed IDE features a coplanar design with an interlaced comb structure. Its mutually interconnected configuration allows it to fit within a small area of the film. The electrode combs are connected to bonding strips at each end, ensuring electrical contact. Figure 1 presents a schematic of the IDE structure.

The electrode consists of 15 pairs with dimensions of 10 mm × 10 mm (L), a line width of 80 µm (H), and a finger gap of 80 µm (G). The surface is coated with 1.0 µm of Au, while the conductive inner layer comprises 5.0–10.0 µm of Cu. The deposited material underwent vacuum drying at 60 °C for 24 h. The effective measurement area remained consistent at 1 cm^2^ across all samples. Normalization was performed with respect to a circular deposition area at the electrode center, defined by a mask of 0.81 cm^2^ with a diameter of 0.51 cm. The deposition volume was precisely controlled using a 0.5–10 µL volumetric pipette, dispensing 0.4 µL per application. All measurements were conducted at room temperature (25 °C).

The electrochemical biosensor comprised working electrodes, reference electrodes, and counter electrodes (WEs, REs, and CEs, respectively). An electrical impedance output (Z) measured in ohms was applied during detection, where the intensity was proportional to the analyte’s activity, allowing for the real-time monitoring and measurement of this activity. EIS is the most efficient and cost-effective analysis technique [9].

## 3. Characterization

### 3.1. Porosity, BET Surface Area, and Isotherm

To characterize the porous structure of BaTiO_3_ nanoparticles and the pore occupancy during albumin functionalization, we used datasets that describe surface and pore characteristics through N_2_ adsorption-desorption analyses; this is also known as the Brunauer-Emmett-Teller (BET) technique. Measurements were conducted using an Accelerated Surface Area and Porosimetry System (Micromeritics 2020 Plus). N_2_ adsorption was performed at −196 °C, with a degassing ramp rate of 10 °C/min, reaching a final temperature of 90 °C. The average evacuation rate was 2.0 mmHg/s, and the relative pressure (P/P_0_) ranged from 0 to 0.1, with an ambient pressure of 760 mmHg. The specific surface area was characterized using the BET equations and devalued before evaluating the BaTiO_3_ samples under calcination at temperatures of 160–340 °C for 10 h.

This section is divided into several subsections. A concise and precise description of the experimental results, their interpretation, and experimental conclusions should be provided.

### 3.2. Molecular Characterization Using FTIR Spectroscopy

Fourier transform infrared (FTIR) spectroscopy was used to identify chemical functional groups. The measurements were performed using a Shimadzu IRPrestige-21 spectrometer equipped with an attenuated total reflectance (ATR) accessory. Powdered BaTiO_3_ samples were analyzed, and specialized software applied spectral enhancements.

### 3.3. Morphological Characterization Using SEM

The surface morphology of mesoporous BaTiO_3_ nanoparticles was examined using scanning electron microscopy (SEM) together with energy-dispersive X-ray spectroscopy (EDS, Thermo Scientific UltraDry). The imaging parameters were as follows: an accelerating voltage of 20 kV, a current of 16 pA, and a spot size of 2.0. Images were acquired at magnifications of ×5 K, ×20 K, ×60 K, and ×100 K. Before imaging, the samples were metalized with an estimated Au-layer thickness of 200 Å at the Laboratory of Analytical Resources and Calibration (LRAC/FEQ), UNICAMP-SP.

### 3.4. Structural Crystallographic and Rietveld (XRD)

The crystallographic phases and material crystallinity were analyzed using X-ray diffraction (XRD) with a Rigaku MiniFlex-600 diffractometer (Rigaku Co., Tokyo, Japan). Cu Kα radiation (λ = 1.54056 Å) was employed, scanning a 2θ range of 10° to 80° with a step of 0.05° and a scan rate of 5° per minute. Diffraction profiles were identified and refined using the HighScore software, V2 version by applying the Rietveld method. The peak height, width, and positions were used to determine the structural parameters and improve theoretical peak profile fitting.

### 3.5. EIS

Complex impedance spectroscopy was used to analyze and characterize the electrical impedance spectroscopy (EIS) results. Because it relies on electrical signals, this technique is nondestructive and does not damage the electrode, allowing it to be washed and reused. The electrochemical behavior of the material under study—including the hybrid system, albumin, BaTiO_3_ nanoparticles, and cortisol—was examined by depositing these components onto the electrode.

To obtain meaningful data from this technique, measurements were conducted using a potentiostat/galvanostat (Metrohm Autolab PGSTAT302n, Metrohm Autolab B.V., Utrecht, Netherlands) equipped with an impedance module and controlled via NOVA software (version 1.11). We employed an arrangement comprising a reference electrode (ER), a counter electrode (CE), and a working electrode (ET). A fixed sinusoidal voltage of 0.5 V was applied over a logarithmically spaced frequency range of 0.01 Hz to 10 kHz. Additional parameters included a sine wave input, a maximum integration time of 1 s, up to three integration cycles, and a current ranging from 100 nA to 1 A. Measurements were performed at an ambient temperature of 25 °C. For data analysis and fitting, this system was represented using circuit models, specifically the Randle model circuit R(RC). A total of 50 data points were acquired per measurement during the impedance spectroscopy, with 300 interaction cycles performed.

## 4. Results and Discussion

### 4.1. Porosity, BET Surface Area, and Isotherm Analysis

**Figure 2:**
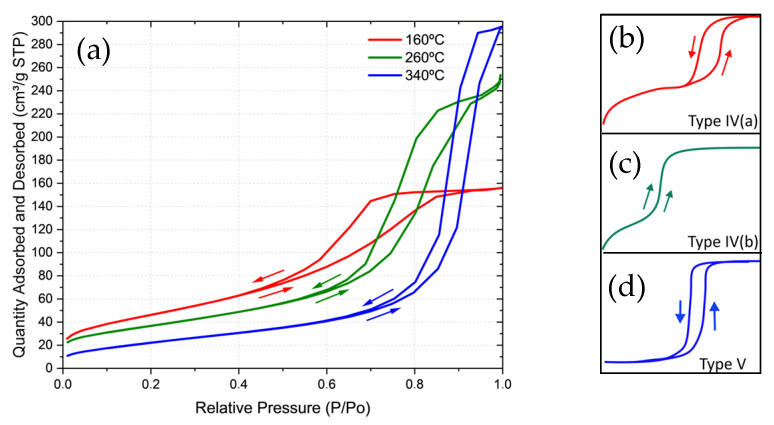
(**a**) A graph showing the relative pressure absorption limits (P/P_0_) and absorbed amount (cm^3^/g STP) for samples calcined at 160 °C, highlighting the temperatures of 160 °C, 260 °C, and 340 °C. For the studied temperatures of 160 °C, 180 °C, 200 °C, 220 °C, 240 °C, 260 °C, 270 °C, 280 °C, 300 °C, and 340 °C, the BET area (cm^3^/g) and pore size (nm) are shown. The insects show the areas of BET type IV (**b**), type IV (**c**), and type V (**d**) with the forms of hysteresis and the classifications identified.

The N_2_ adsorption-desorption isotherms of the 160, 260, and 340 °C samples (Figure 2a), and the Barrett-Joyner-Halenda (BJH) pore-size distributions were analyzed. The hysteresis phenomenon suggested the involvement of type IVa, IVb, and V behaviors, respectively (Figure 2b–d). These classifications align with the physisorption isotherms of mesoporous materials, as defined by the IUPAC classification [10], within the relative pressure range of 0–1.0 P/P_0_, obtained using N_2_ at 77 K.

The type IVa isotherm was identified at the calcination temperature of 16 °C, where hysteresis was observed. This is associated with capillary condensation, which occurs when the pore width exceeds a critical threshold, transitioning from a hydrophilic to a hydrophobic state. At 260 °C, the material exhibited a type IVb isotherm, characterized by mesopores of smaller width. In this case, the adsorption behavior was nearly reversible and attributed to conical and cylindrical mesopores that were closed at their tapered ends. At 340 °C, the material exhibited a type V isotherm associated with relatively weak adsorbent-adsorbate interactions, particularly for water adsorption on hydrophobic microporous and mesoporous adsorbents. These findings indicate that the calcination temperature affects the adsorption system and overall adsorption behavior.

The BET and pore diameter values at 170.91 (m^2^/g) were considered large, especially for a material such as BaTiO_3_ at 5.24 nm, 133.77 (m^2^/g), and 10.33 nm, 86 (m^2^/g), and 17.92 nm [11].

The adsorption plot exhibits a low-slope region associated with multilayer adsorption on the pore walls, followed by mesopore condensation. It concludes with a plateau region, indicating that the mesopores have limited or nonexistent macroporosity. The desorption plot features a narrow hysteresis loop, where the desorption branch runs parallel to the adsorption branch. This parallel behavior suggests a narrow distribution of uniform mesopores and minimal networking effects—characteristics associated with type H1 hysteresis.

The isotherm in Figure 2a suggests that the synthesized BaTiO_3_ possesses a stable mesoporous structure, with adsorption decreasing as temperature increases—a trend expected for mesoporous materials. SEM images reveal that the 160 °C sample has a highly porous morphology, corresponding to large particles, as confirmed by BET measurements. These structural characteristics are particularly beneficial for optimizing the mesoporous structure of BaTiO_3_ in applications where adsorption and desorption properties are critical. This is especially relevant in applications requiring precise control over the surface area and pore distribution, which directly influence functional properties in electrochemical devices such as the cortisol sensor.

Furthermore, specific surface area (BET) data from our BaTiO_3_ synthesis (Table 1) indicate a reduction in the surface area with increasing calcination temperature [1,12]. At 160 °C, the specific surface area is relatively high (170.91 m^2^/g), whereas at 340 °C, it decreases to 86.85 m^2^/g. Additionally, the pore size increases from 5.24 to 17.92 nm at 340 °C—a trend corroborated by previous studies.

The isotherm graph, which plots relative pressure absorption limits (P/P_0_) against the absorbed amount (cm^3^/g STP) for samples calcined at 160 °C, suggests that the synthesized BaTiO_3_ has a stable mesoporous structure. As expected for mesoporous materials, increasing the calcination temperature reduces the amount of absorption. Table 1 presents the BET and pore-size values for samples calcined at temperatures ranging from 160 to 340 °C. SEM images reveal that the 160 °C sample has a highly porous morphology, corresponding to the presence of large particles, as confirmed by the BET measurements. These structural characteristics are particularly beneficial for optimizing the mesoporous structure of BaTiO_3_ in applications where absorption and desorption properties are critical. This is especially relevant for applications requiring precise control over surface area and pore distribution, as these factors directly influence the functional properties of BaTiO_3_ in electrochemical devices such as the cortisol sensor [10].

### 4.2. Molecular Characterization (FTIR)

**Figure 3:**
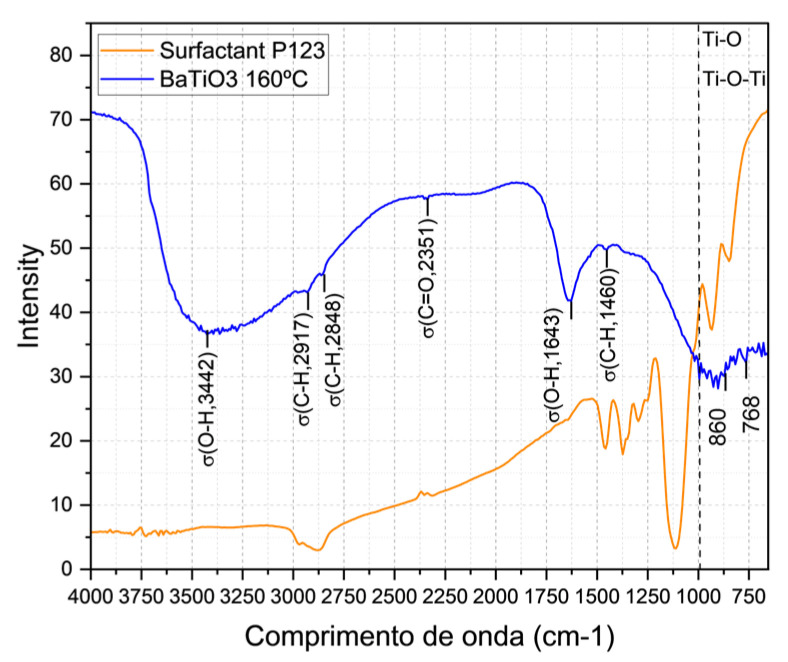
FTIR spectra obtained with the KBr pasting of the surfactant Pluronic^®^ P-123; the sample heated at 160 °C for 10 h, and the absence of the contribution of the P123 surfactant in the wavelength and absorption spectrum of BaTiO_3_, which disappeared at around 2800–3000 cm^−1^ and in the bands of 1150–1400 cm^−1^.

Molecular characterization was performed using vibrational spectroscopy at a calcination temperature of 160 °C. The results confirmed the presence of Ti-O and Ti-O-Ti bonds [13], characterizing the nanoparticles. The properties of BaTiO_3_ were maintained, showing suitable porosity to function as an adsorbent. The surfactant Pluronic^®^ P123 disappeared in the 2800–3000 and 1150–1400 cm^−1^ bands, indicating that it was removed after calcination. These bands were attributed to the σ(C-H) and σ(C-O-C,1080) elongation vibrations of P123, respectively [14], as shown in Figure 3.

### 4.3. Morphological Characterization (SEM)

**Figure 4:**
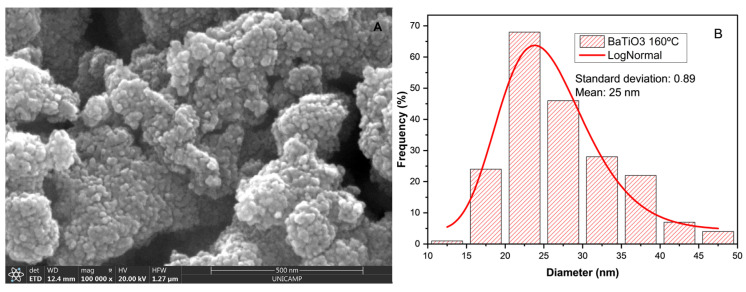
(**A**) The SEM image of BaTiO_3_ nanoparticles at 100,000× magnification; measurements were obtained from samples that were calcined at 160 °C; (**B**) histogram of the measured sample sizes for the calcination temperature of 160 °C.

The formation of smaller primary particles with an average size of 26 nm was observed. We consider this a promising morphology for the sensor presented in Figure 4, which refers to the sample at 160 °C.

To determine the average size of the primary particles (25 nm), 200 measurements were conducted, and a histogram was generated. This histogram illustrates the frequency distribution of the particle-size measurements for the sample calcined at 160 °C, as shown in Figure 4, providing a detailed characterization of the nanoparticles.

### 4.4. Structural, Crystallographic, and Rietveld Analysis (XRD)

The 01-073-1764 (anatase), 00-029-1360 (brookite), and 01-088-1173 (rutile) standards were imported into the appropriate software. The coordinates of the atomic structure used for TiO 5000223 are given: (parameters a = 3.7892(4) Å, b = 3.7892(4) Å, c = 9.537(1) Å, α = 90°, β = 90°, γ = 90°).

**Figure 5:**
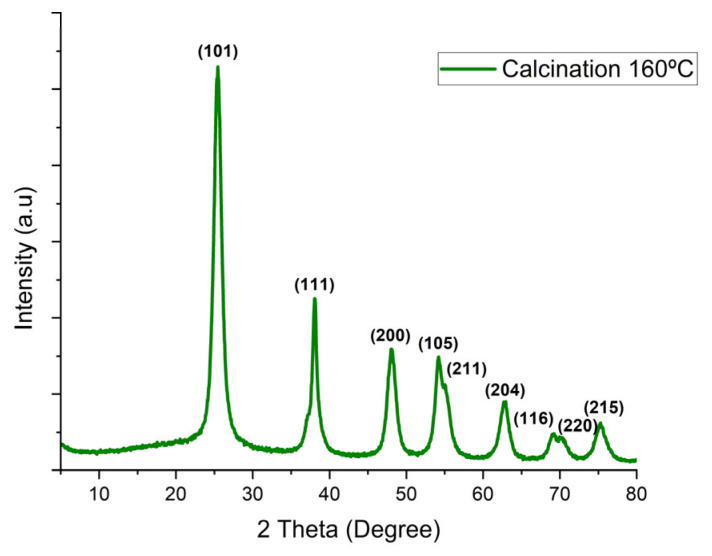
XRD pattern of the sample calcined at 160 °C, exhibiting characteristic TiO_2_ crystalline peaks. Additionally, we analyzed the effects of the calcination temperature (ranging from 160 to 340 °C) on the phase morphology and crystallite size using Rietveld refinement [15].

The crystallographic profiles of the sample calcined at 160 °C, which was identified as the optimal calcination temperature, were analyzed using XRD. The observed diffraction peaks at 25.49°, 38.05°, 48.17°, 54.13°, 55.18°, 62.84°, 68.85°, 70.34°, and 74.30° correspond to the (101), (111), (200), (105), (211), (204), (116), (220), and (215) crystalline planes, respectively. These peaks indicate a dominant anatase phase, which aligns with the standard TiO_2_ reflections identified through Miller indices in various studies (see Figure 5).

The effect of thermal treatment on morphology was related to the temperature and particle size. An increase in temperature corresponds to a slight increase in the anatase phase, a slight decrease in the brookite phase, and a gradual increase in the crystallite size, as shown in Table 2.

### 4.5. Cortisol Detection Using Complex Impedance Spectroscopy

Standard analytical cortisol samples were prepared at 5.0, 7.5, 10, and 20 ng/mL concentrations. The complex impedance was recorded using a constant sinusoidal input voltage of 5 mV over the frequency range of 0.01 Hz to 10 kHz, with a nominal current of 40 nA. Measurements were conducted using a Metrohm^®^ Autolab PGSTAT302n. EIS was employed to evaluate the sensor’s performance parameters, with cortisol levels analyzed at the optimal calcination temperature of 160 °C.

**Figure 6:**
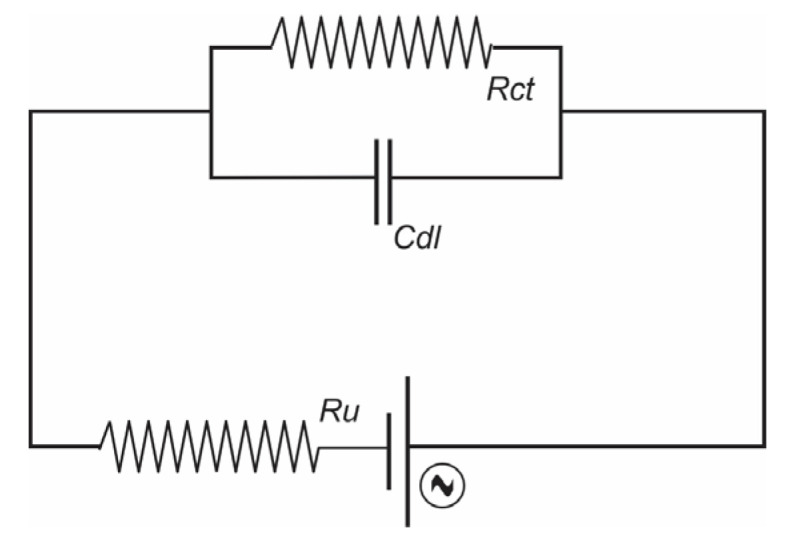
Simplified Randle’s typical circuit.

The results are presented as a Nyquist plot. The real component of the complex impedance (Z′) and the complex impedance imaginary component (Z″), measured in ohms, were plotted against the cortisol concentration. In our electrochemical system, the Nyquist graph displays faradaic impedance spectra over a wide frequency range, including a semicircle corresponding to the frequency region where charge-transfer phenomena govern the electrochemical process. Using equivalent electrical circuits that can contain quantities such as capacitance (C), resistance (R), and/or inductance (L), we represent the processes related to real (Z′) and imaginary (Z′) impedances. This system can be represented by circuit models. We performed analysis and fitting adjustments using the Randle model circuit R(RC) shown in Figure 6 [9].

**Figure 7:**
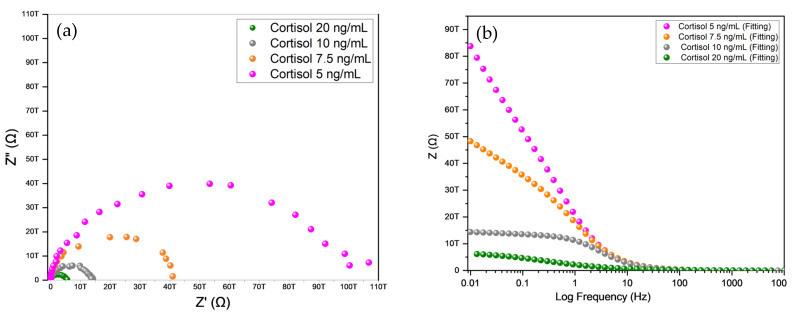
(**a**) The charge-transfer resistance (Ru) corresponded to the diameter of the Nyquist plot and increased with the cortisol concentration. The increase in Ru was attributed to cortisol binding to the hybrid system, albumin, and deposited BaTiO_3_ nanoparticles, producing an insulating layer that resisted charge transfer from the solution to the electrode. The cortisol concentration of 5 μg/mL presents the largest arc with the highest Z′ value of approximately 110 TΩ; the curve of 7.5 μg/mL has a value of 45 TΩ; the 10 μg/mL arc has a value of 20 TΩ; and 20 μg/mL exhibits the smallest arc of 10 TΩ. (**b**) A Bode plot with frequency spectra (Hz) and logarithmic scale total impedance (Z) in Ω for the hybrid system, albumin, BaTiO_3_ nanoparticles deposited on the electrode and measured at five cortisol concentrations of 5.0, 7.5, 10, and 20 ng/mL and fitting components on the Randle circuit R(RC).

The Nyquist graph shown in Figure 7a portrays the spectra of real impedance (Z′) versus complex impedance (Z″) in Ω for the hybrid system, albumin, and BaTiO_3_ nanoparticles deposited on the electrode and measured at five concentrations of cortisol: 5.0, 7.5, 10, and 20 ng/mL, and fitting components measured on the Randle circuit R(RC) measured. The impedance spectra were measured on the IDE after each modification process. In these spectra, the diameter of the semicircle represents the charge-transfer resistance (Rct) on the IDE surface of the sensor solution after the charge resistance. The deposited hybrid system comprised 0.2 g of material combined with 0.1 mL of a dilute NH_4_OH solution, albumin, 0.4 mL of absolute alcohol, 0.2 mL of Triton, and 0.01 g of BaTiO_3_ nanoparticles. This mixture was measured at four cortisol concentrations (0.3 µL) and vacuum-dried at 60 °C for 24 h before analysis.

With the increase in cortisol concentration (from 5 to 20 μg/mL), there was a significant reduction in the Z′ value, indicating a decrease in the electrochemical system’s charge-transfer resistance (Rct). This suggests that the sensor is sensitive to the cortisol concentration and exhibits higher conductivity (lower impedance). These concentrations facilitated charge transport in the system, indicating the more efficient and consistent electrochemical response of the sensor and showing that it can distinguish different cortisol levels according to the impedance variation [16].

The analysis of the data in the Bode plot (Figure 7b) provided information on the phase response of the system at different cortisol concentrations, such as the electrochemical response of the sensor to cortisol. The results of the Bode plot, which display cortisol concentrations of 5.0, 7.5, 10, and 20 ng/mL, were analyzed using a fitted Randle circuit model R(RC). Table 3 presents the correlations between the calculated cortisol concentrations and the corresponding values of the solution resistance (Ru), charge transfer (Rct), and double-layer capacitance (Cdl), along with the estimated error Z′ and Chi-squared value. When measuring each concentration on the other hand, linear behavior at lower frequencies indicated the presence of a purely capacitive component characterized by angles close to 90 °C and was plotted with frequency (Hz) versus total impedance (Z) in Ohms (Ω), and frequency (Hz) versus the phase angle (Ø) in degrees, using a logarithmic scale for frequency (Hz) and a linear scale for the phase angle (Ø) [9].

The phase angle (Ø) of the impedance (Z) varied across frequency ranges, increasing at medium frequencies before stabilizing again at high frequencies. At low frequencies, the phase was approximately 0° for most concentrations, indicating that the system was predominantly resistive with the current and voltage in phase. At medium frequencies, the phase increased, reaching a peak that signified the presence of charge-storage processes such as double-layer capacitance (Cdl). The phase began to stabilize at high frequencies again, indicating the predominance of processes such as solution resistance (Ru) (Table 3) [9].

For the cortisol concentration of 5 ng/mL, there was a sharp increase in the phase, reaching a peak close to 65°, indicating a more pronounced capacitive response at mid-frequencies. At 7.5 ng/mL, the phase started lower and reached a reduced peak of approximately 55°, suggesting a less intense capacitive response than 5 ng/mL. The phase was intermediate for 10 ng/mL, with a peak at around 60°, suggesting a balance between resistance and capacitance at mid-frequencies. It was consistently lower for 20 ng/mL, reaching a maximum peak of approximately 45° and indicating greater relative resistance and less capacitance compared with the other concentrations.

As the cortisol concentration increased, the phase decreased, indicating that cortisol may have formed a more compact layer on the electrode surface, reducing the capacitive contribution and increasing the dominance of the resistive response.

It is also evident that the frequency response (Hz) of the IDE equivalent circuit, which is the total impedance Z, is dominated by the different circuit elements at different frequencies. The Bode plot provided a complementary perspective to the Nyquist plot, revealing that higher cortisol concentrations resulted in lower total impedance (Z). The Bode plot with frequency spectra (Hz) and logarithmic scale total impedance (Z) in Ω measured the hybrid system, albumin, and BaTiO_3_ nanoparticles deposited on the electrode at five concentrations of cortisol 5.0, 7.5, 10, and 20 ng/mL [16].

### 4.6. Electrochemical Analysis Using Salivary Samples

**Figure 8:**
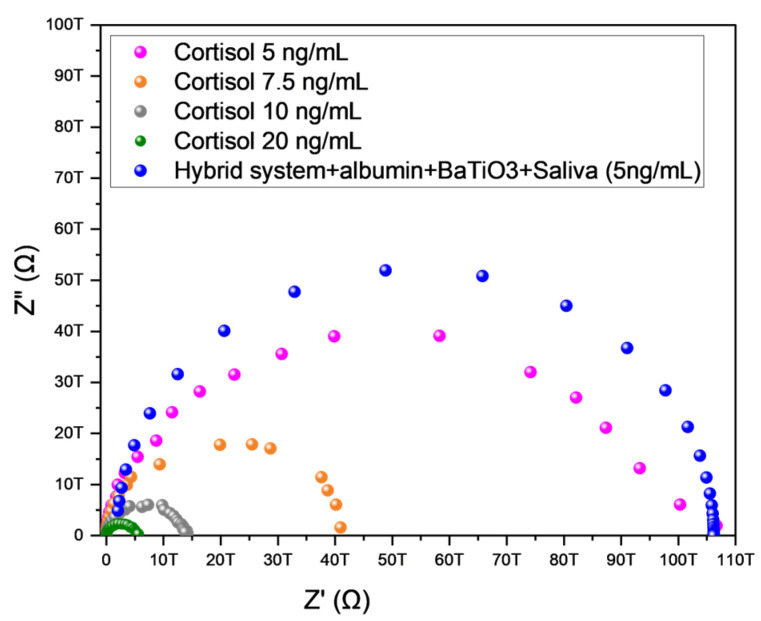
Nyquist plots obtained via EIS using an IDE modified with mesoporous BaTiO_3_ nanoparticles for the detection of cortisol in a saliva sample quantified at 5 ng/mL exhibited a clear semicircle in the high-frequency region, corresponding to the charge-transfer resistance (Rct), which expanded upon cortisol binding. These results confirm the effective detection of low cortisol concentrations in biological fluid coinciding with the typical physiological range of salivary cortisol (~5.27 ng/mL).

In the preliminary approach, an electrochemical measurement was performed using a sensor functionalized with a hybrid sensing layer comprising mesoporous BaTiO_3_ nanoparticles, BSA, and an organic-inorganic urethane-siloxane polymer. This configuration was deposited onto IDEs, establishing a sensitive and selective electrode-sample interface for cortisol detection in saliva. The electrochemical response illustrated in Figure 8 was obtained through EIS using this hybrid functional layer. The architecture promoted effective biomolecular interaction and facilitated an efficient electrochemical signal. Notably, the sensor exhibited a distinct and consistent electrochemical response at a cortisol concentration of 5 ng/mL, aligning closely with the typical physiological salivary cortisol level (~5.27 ng/mL) (Vignesh, 2024 [2]). This result demonstrates the sensor’s capability to operate not only under controlled laboratory conditions but also within the biologically relevant concentration ranges found in human samples. The observed decrease in impedance (Z′) at this concentration confirms the system’s sensitivity, suggesting that the combination of mesoporous BaTiO_3_ takes place, with its large surface area and elevated dielectric constant; BSA as a stabilizing agent and urethane-siloxane as a flexible matrix constitute an effective platform for selective cortisol detection. The sensor’s reliable performance within the physiological range indicates its potential for integration into point-of-care technologies aimed at real-time stress monitoring and endocrine health assessment.

## 5. Conclusions

Mesoporous BaTiO_3_ nanoparticles were synthesized via the sol-gel method and integrated into a hybrid sensing matrix containing albumin. This study demonstrates that the cortisol sensor based on an IDE platform functionalized with BaTiO_3_ nanoparticles has considerable potential for the noninvasive, real-time monitoring of cortisol levels. Comprehensive morphological, molecular, and crystallographic characterizations verified the quality and functional integrity of the mesoporous BaTiO_3_ structure within the electrochemical interface. The synthesis strategy allowed control over physicochemical properties, as confirmed by BET analysis, which revealed characteristic hysteresis loops across calcination temperatures ranging from 160 °C to 340 °C. Notably, calcination at 160 °C yielded a large specific surface area and optimal pore distribution. These characteristics increased the adsorption capacity, which is critical for sensor performance. The shift in hysteresis behavior with an increasing temperature, from type IV(a) to IV(b) and V, reflects a transition from hydrophilic to hydrophobic surface properties. EIS measurements confirmed the sensor’s responsiveness to cortisol, with a consistent reduction in real impedance (Z′) correlated with increasing hormone concentrations. Importantly, Nyquist plots obtained for a saliva sample containing 5 ng/mL cortisol—aligned with the typical physiological salivary range—exhibited a distinct increase in charge-transfer resistance (Rct), confirming the system’s capacity to detect clinically relevant hormone levels in biological matrices. These findings demonstrate that the proposed sensor architecture integrates high sensitivity, reproducibility, and practicality, offering a robust platform for point-of-care diagnostic applications targeting stress-related biomarkers.

## Figures and Tables

**Table 1:** BET surface area and pore size for samples calcined at different temperatures.

Calcination Temperature (°C)	BET (m^2^/g)	Pore Size (nm)
160 °C	170.91	5.24
180 °C	158.81	5.47
200 °C	133.46	7.46
220 °C	143.26	9.05
240 °C	144.18	10.49
260 °C	133.77	10.33
270 °C	91.20	13.66
280 °C	89.51	14.23
300 °C	95.20	14.69
340 °C	86.85	17.92

**Table 2:** The effects of the calcination temperature (160–340 °C) on the phase morphology and crystallite size with a calcination time of 10 h determined using Rietveld analysis.

Calcination Temperature (°C)	Crystalline Phase (%)			Crystallite Size (nm)		
	Anatase	Brookite	Rutile	Anatase	Brookite	Rutile
160 °C	63.7	36.3	0	46.6	6.8	0
180 °C	66.8	33.2	0	51.6	11.8	0
200 °C	69.9	30.1	0	56.6	13.1	0
220 °C	73.0	27.0	0	61.6	14.1	0
240 °C	76.1	23.9	0	66.5	22.1	0
260 °C	79.2	20.8	0	71.5	30.1	0
270 °C	82.5	17.5	0	95.0	39.1	0
280 °C	82.5	17.5	0	95.0	39.1	0
300 °C	92.9	7.1	0	77.5	66.3	0
340 °C	92.6	7.3	0	84.9	64.8	0

**Table 3:** Correlations between the measured cortisol concentrations: Ru, Rct, calculated Cdl, estimated error Z′, and Chi-square.

Cortisol ng/mL	Ru (TΩ) Measured	Rct (TΩ) Measured	Cdl (F) Calculated	Estimated Error Z′ (%)	Chi-Square (X^2^)
5	169	92	0.017917	3.62	0.72
7.5	178	41	0.014632	5.25	0.19
10	183	14	0.014211	6.65	0.66
20	185	6	0.009717	8.05	0.84

## Data Availability

Data are contained within the article.
